# Pollen Grain Classification Using Some Convolutional Neural Network Architectures

**DOI:** 10.3390/jimaging10070158

**Published:** 2024-06-28

**Authors:** Benjamin Garga, Hamadjam Abboubakar, Rodrigue Saoungoumi Sourpele, David Libouga Li Gwet, Laurent Bitjoka

**Affiliations:** 1 ENSAI, Laboratory of Energy, Signal, Imaging and Automation, University of Ngaoundere, Ngaoundere P.O. Box 455, Cameroon; benjamin.garga@univ-ndere.cm (B.G.); rsaoungoumi@univ-ndere.cm (R.S.S.); david.libouga@univ-ndere.cm (D.L.L.G.); lbitjoka@univ-ndere.cm (L.B.); 2 Departement of Computer Engineering, University Institute of Technology, University of Ngaoundere, Ngaoundere P.O. Box 455, Cameroon; 3 Laboratory of Analysis, Simulations and Tests, University of Ngaoundere, Ngaoundere P.O. Box 455, Cameroon; 4 ENSAI, Department of Mathematics and Computer Sciences, University of Ngaoundere, Ngaoundere P.O. Box 455, Cameroon; 5 EGCIM, Department of Fundamental Sciences and Engineering Techniques, University of Ngaoundere, Ngaoundere P.O. Box 455, Cameroon; 6 ENSAI, Department of Electrical Engineering, Energetics and Automation, University of Ngaoundere, Ngaoundere P.O. Box 455, Cameroon

**Keywords:** pollen grains, classification, performances, CNN, dataset, POLLEN73S, 35A01, 65L10, 65L12, 65L20, 65L70

## Abstract

The main objective of this work is to use convolutional neural networks (CNN) to improve the performance in previous works on their baseline for pollen grain classification, by improving the performance of the following eight popular architectures: InceptionV3, VGG16, VGG19, ResNet50, NASNet, Xception, DenseNet201 and InceptionResNetV2, which are benchmarks on several classification tasks, like on the ImageNet dataset. We use a well-known annotated public image dataset for the Brazilian savanna, called POLLEN73S, composed of 2523 images. Holdout cross-validation is the name of the method used in this work. The experiments carried out showed that DenseNet201 and ResNet50 outperform the other CNNs tested, achieving results of 97.217% and 94.257%, respectively, in terms of accuracy, higher than the existing results, with a difference of 1.517% and 0.257%, respectively. VGG19 is the architecture with the lowest performance, achieving a result of 89.463%.

## 1. Introduction

Pollen grains are tiny particles carried by the wind, and are essential to the reproduction of flowering plants. They are produced by the male organs of flowers, called stamens, in particular the anthers, which contain the pollen grains. Pollinators in general ensure the reproduction of flowering plants, contributing to biodiversity and playing a crucial role in climate regulation, although climate change may disrupt this synergy [1]. The classification of pollen grains represents a major challenge in the fields of plant biology and paleoecology (paleoecology is the study of the complex relationships between living organisms, their physical environment and their evolution over time, using fossil, archaeological and sedimentological data.) This process is of great importance, as it enables the identification of the plants responsible for these grains and the reconstruction of environmental history through the ages thanks to sediment analysis [2,3].

Pollen analysis is also widely used to detect and monitor allergenic particles in the air. The duration of pollen seasons has grown longer in recent years due to the effects of global warming and changes in climate conditions [4]. As a result, individuals who are exposed to high levels of allergenic pollen in the air have experienced an increase in seasonal allergies [5]. The recognition of pollen grains in palynological investigation is crucial for the development of effective treatments for patients with allergic rhinitis. By employing this method, patients and healthcare providers can track the level of allergenic pollen present in the air, plan their outdoor activities accordingly, and manage their medical treatments accordingly [6]. The study of pollen grains present in individuals, objects, air, pollinators and beekeeping products also contributes to the protection, surveillance and monitoring of flora in order to preserve this ecosystem [4].

The creation of image datasets that contain numerous examples categorized by experts is crucial for automating pollen grain analysis. The task requires a lot of hard work and specialized equipment, like an optical microscope and slides. The lack of pollen grain image datasets specifically designed for computer vision automation is a result of this reason [4]. The training of efficient convolutional neural networks (CNNs) can be hindered by a limited number of examples, which could result in the poor performance of learning models [4]. CNNs are deep neural networks capable of learning complex patterns from images. They have been used successfully in the classification of pollen grain images. Using these networks, it is possible to accurately classify images of pollen grains from different species.

The main goal of this work is to improve the performance of eight (08) architectures of Astolfi et al. [4] for the classification of Pollen73S, using the holdout cross-validation method according to the accuracy [7,8]. The work is structured as follows: Section 2 reviews the state of the art in pollen grain classification using CNN. Section 3 describes the POLLEN73S dataset of Astolfi et al. [4] used in this work. An overview of the methods used is provided in Section 4. Results and discussions are the focus of Section 5. The conclusion rounds up the paper.

## 2. Literature Review of Related Work

Pollen grain classification has been a focus for several state-of-the-art convolutional neural networks (CNNs) in recent years. Daood et al. developed a seven-layer deep convolutional neural network, as described in [9], that underwent training on a dataset comprising 30 classes of pollen grains, achieving a correct classification rate of 94%. A dataset with 46 different pollen grain classes was classified by pretrained AlexNet in [10]. The incorporation of data augmentation and cross-validation techniques resulted in a precision of 98%. To categorize five classes of pollen grains using 13,000 images, AlexNet and SmallerVGGNet were implemented in [11]. Precision rates of 89.63% and 89.73%, respectively, were achieved by both networks. A pollen dataset with 73 pollen grain categories was analyzed by Astolfi et al. in [4]. Eight state-of-the-art CNNs were compared in terms of performance, including Inception V3, VGG16, VGG19, ResNet 50, NASNet, Xception, DenseNet 201 and Inception ResNet V2. They showed that DenseNet201 and ResNet50 outperformed other CNNs (Inception V3, VGG16, VGG19, NASNet, Xception and Inception ResNet V2) with an precision of 95.7% and 94.0%, respectively. Li et al. in [6] analyzed two categories of the Urticaceae family, named Parietaria and Urtica, showing strong morphological similarities composed of three categories of a dataset from 6472 images of pollen grains. To find a better classifier, they used both machine learning and deep learning methods. For the first method, they measured both texture and moment characteristics based on images of pollen grains with feature selection techniques and a hierarchical strategy. Secondly, they compared the performance of six popular CNNs: VGG16, AlexNet, ResNet50, VGG19, MobileNet V1 and MobileNet V2. Machine learning-based methods achieved high precision of 94.5% and deep learning-based methods achieved 99.4%.

In addition to CNN, there are other works that use computer vision techniques as references. Treloar et al. [12] gave us access to a dataset that consisted of 12 pollen grain types, with a handful of grayscale pictures per category collected on Henderson Island, Polynesia. For establishing a baseline with the dataset, the researchers employed a method where geometric characteristics of pollen grains like perimeter, roundness, and surface area were utilized as input to generate texture features. Additionally, a classification technique based on Fisher’s linear discriminant was employed for classification purposes. Depending on the subset of variables used, the proportion of pollen correctly classified can range from 81% to 100%, according to their report. An average classification rate of around 95% was achieved by the best set of variables. In [13], Travieso et al. used a dataset of 564 images. A total of 22 plant species in Costa Rica, Central America were utilized to capture these images. They categorized 47 distinct types of pollen grains using the techniques of the hidden Markov model (HMM) and support vector machines (SVM). An average classification accuracy of 93.8% was achieved by the proposed method. The classification of pollen grains was performed with an HMM classifier by García et al. [14], but they used a method relying on binarization and edge identification to obtain features from the grains. In Costa Rica, Central America, there are 11 distinct tropical honeybee plant families with 426 pollen grains from 17 types in the constructed dataset. An average of 98.77% was achieved in classification by the proposed method. A baseline was established using the same dataset as [14] by Ticay-Rivas et al. [15]. One approach suggested by the researchers includes merging geometric parameters, Fourier descriptors of morphological features utilizing the discrete cosine transform (DCT), and color data to identify distinctive attributes of pollen grains. These attributes are utilized to educate a multilayer neural network classifier, resulting in an average accuracy rate of 96.49%.

Instead of deep learning methods that automatically extract features from the image [9,10,16,17,18,19], machine learning methods are employed to manually select and extract features with specific functions from images [20,21]. Machine learning techniques use handcrafted features based on pollen grain images’ shape, texture, and other related properties. Classification performance is greatly influenced by the extracted features. The use of proper feature selection methods and classifiers is also important for machine learning-based classification methods.

The authors suggested a fusion of geometric and textural characteristics in [22] as distinctive intrinsic attributes for a pollen dataset containing 17 classes. Integrating linear discriminant analysis (LDA) and least squares support vector machines (LS-SVM) led to the optimal performance, achieving an precision of 94.92%. Marcos et al. [23] derived four texture attributes, comprising gray-level co-occurrence matrices (GLCM), log-Gabor filters (LGF), local binary patterns (LBP), and Tchebychev discrete moments (DTM), from a set of pollen images encompassing 15 classes. Subsequently, Fisher’s discriminant analysis (FDA) and K-nearest neighbor (KNN) were utilized for dimensionality reduction and multivariate classification purposes. This resulted in achieving a 95% accuracy rate. Manikis et al. [21] leveraged texture attributes derived from GLCM and seven geometric properties computed from the binary mask of a pollen image collection. Subsequently, a random forest (RF) classifier was employed for classification, obtaining an accuracy of 88.24% for six pollen classes. The outcomes of machine learning exhibit considerable variability, seemingly influenced by the dataset under consideration.

A recent investigation conducted by Djoulde et al. [24] centered on the categorization of pepper seeds through the utilization of color filter array (CFA) images. The research specifically delved into Penja pepper, a variant of *Piper nigrum* predominantly cultivated in the Litoral region of Cameroon. Despite its relatively modest production levels in comparison to larger producers like India and Brazil, Penja pepper is highly esteemed in the market due to its unique quality. The primary objective of this study was to address the challenge individuals face in discerning between various bell pepper types based solely on seed appearance. Through the collection and analysis of 5618 samples encompassing white and black Penja peppers alongside other varieties, the researchers applied image processing techniques and a supervised machine learning approach to extract 18 attributes from the visuals and employ diverse models for classification. The outcomes revealed that the support vector machine (SVM) emerged as the top-performing model, achieving an accuracy rate of 0.87, a precision score of 0.874, a recall of 0.873 and an $F1_{score}$ of 0.874. This study thus represents a significant advancement in the realm of pepper seed classification, integrating cutting-edge image analysis and machine learning methodologies to facilitate the differentiation among various pepper cultivars. Other works on pollen classification using machine learning can be founded in [25,26,27,28,29].

## 3. Description of Dataset POLLEN73S

Astolfi et al. in [4] developed a dataset called POLLEN73S containing annotated images of pollen grains present in the Brazilian savannah. This dataset includes 2523 images of pollen grains from 73 different types, captured at various angles. The plant species in bloom, from which these pollen grains were sourced, were gathered within a 1.5 km distance from coordinates 20°23′16.8″ S 54°36′36.3″ W, situated in the urban vicinity of Campo Grande City, the capital of the state of Mato Grosso do Sul in Brazil.

**Figure 1:**
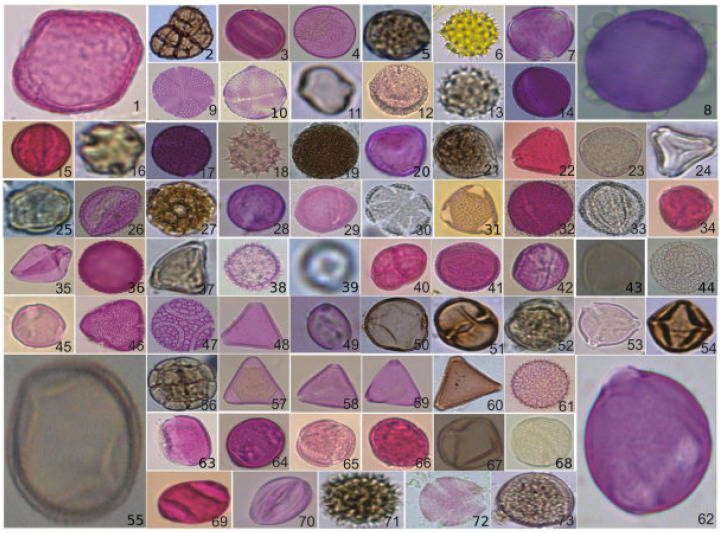
The sampling process for the POLLEN73S dataset (see [4] for the name of each image).

The dataset POLLEN73S comprises reduced images obtained by resizing larger images. Given the varying sizes of the pollen grains, the image dimensions in the POLLEN73S dataset exhibit diversity. Specifically, 88.6% of images possess an average size of 512 × 512 pixels or less, with the remaining 11.4% exceeding this measurement. The images were resized to 224 × 224, which is the standard input size used for all the trained models mentioned in Section 4.2. This size was chosen to ensure compatibility with already pretrained models, maintain a balance between quality and performance, and simplify the process in computer vision. In Figure 1, an exemplar of a pollen grain is displayed for each category. Every category contains 35 images, except for Gomphrena sp. with 10 images, Trema micrantha with 34 images, and Zea mays with 29 images [4]. POLLEN73S offers an exhaustive variety of pollen grains, providing satisfactory categorical coverage and diversity of examples, to promote the progress of computer vision in the field of palynology.

## 4. Methods

### 4.1. Block Diagram of the Proposed Methodology

**Figure 2:**
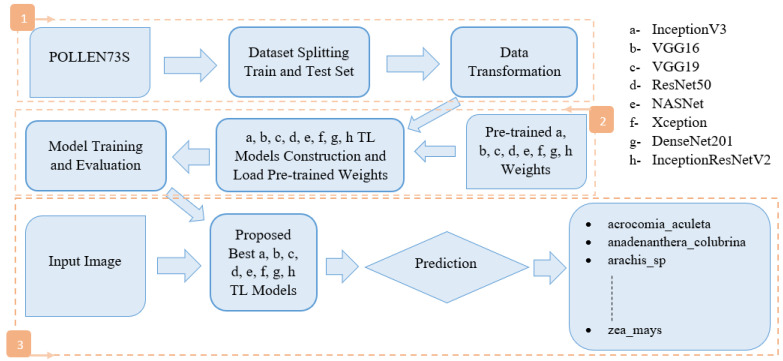
A block diagram of the proposed methodology.

Figure 2 depicts the methodology used in this work.

We divided the POLLEN73S dataset into two distinct sets: a training set and a test set. Section 4.3 outlines the importance of using the training set to train the model and the test set to validate and test it. After that, we applied data augmentation, dropout layers, fine tuning and hyperparameters to the training dataset (Section 4.4). We then set up transfer learning models. The TL models designed (a, b, c, d, e, f, g and h as shown in the Figure 2, which we will detail in Section 4.2), as well as the loading of pretrained weights, model training and evaluation are carried out. Finally, we subjected the input image to each best TL model proposed, and proceeded with prediction to produce the expected result, namely the percentage of membership of each class.

### 4.2. CNN Architectures for Pollen Grain Classification

A transfer learning approach was implemented to overcome the limitations of the small training dataset and avoid overlearning using ImageNet’s pretrained weights. In this study, the final layer of seven CNN models—VGG16, VGG19, DenseNet12, InceptionResNetV2, InceptionV3, ResNet50 and Xception—was tuned while exploiting the pretrained model only as a feature extractor. To adapt these models to our classification problem, the final layers comprising fully connected layers with softmax activation were replaced by a flattened layer for data transformation, a dropout was introduced for regularization, as well as hyperparameters to control the learning process. In addition, a dense layer has been included to apply softmax activation and generate probabilities for the classes associated with “POLLEN73”.

#### 4.2.1. Inception V3

**Figure 3:**
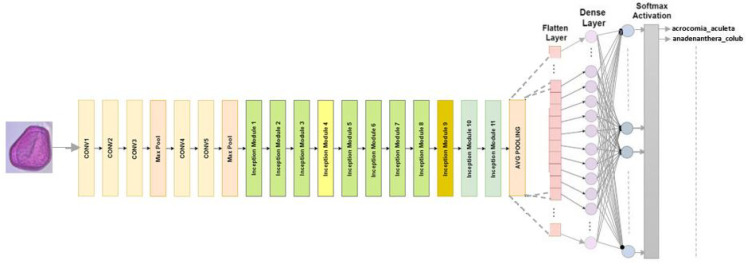
InceptionV3 architecture designed for multiclass classification.

The classification we use it for consists of 48 layers with 11 creation modules. The image input size for InceptionV3 is 299 × 299. The ReLU activation function and convolution filters, clustering layers, and other elements comprise every module [30]. To reduce the number of parameters without compromising network efficiency, convolutions are factorized. The Figure 3 shows the refined InceptionV3 model used for our classification.

#### 4.2.2. VGG 16

**Figure 4:**
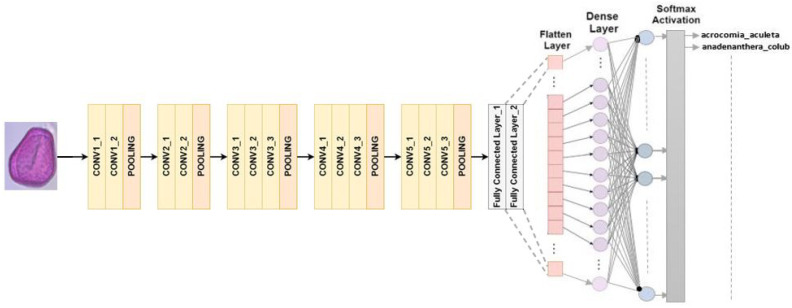
VGG16 architecture for multiclass classification.

RGB images can be processed with a set input size of 224 × 224 pixels in the VGG16 architecture. A total of 13 convolutional layers and 3 fully connected layers are the main components of the composition that consists of 16 layers. The final classification process involves using max-pooling to decrease the volume size, and using softmax activation in the last fully connected layer [30]. In this study, we replaced the last fully connected layer with softmax activation with our own classifier, as shown in Figure 4.

#### 4.2.3. VGG 19

**Figure 5:**
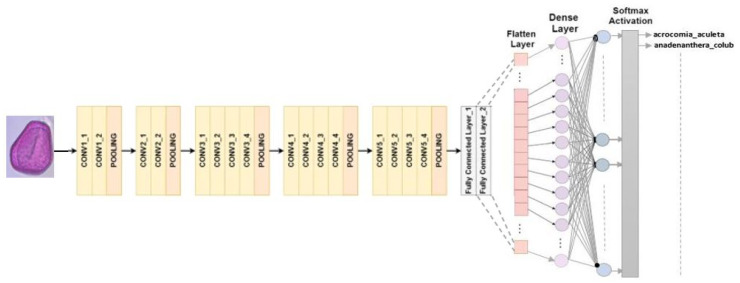
VGG19 architecture for multiclass classification.

There are 19 layers in the structure, with 16 convolutional layers and three fully connected layers. The volume size can be reduced by using max-pooling, and classification is carried out using the last fully connected layer with softmax activation [30]. In this study, we substituted our own classifier for the last fully connected layer with softmax activation, as illustrated in Figure 5.

#### 4.2.4. ResNet 50

**Figure 6:**
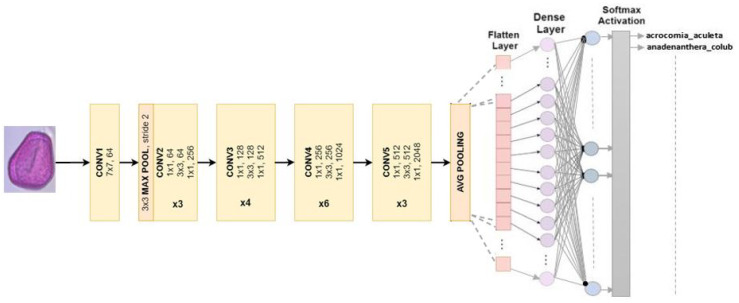
Multiclass classification is the intended use for ResNet50 architecture.

ResNet, or the Residual Network, is modified into a different version. The arrangement is comprised of 49 convolution layers, a max pool layer, and a medium pool layer. There are three convolution layers in every convolution block, and there are also three convolution layers in every identification block. ResNet50 has over 23 million adjustable parameters [30]. Figure 6 shows our adapted ResNet50 model used to classify Pollen73s.

#### 4.2.5. NASNet

**Figure 7:**
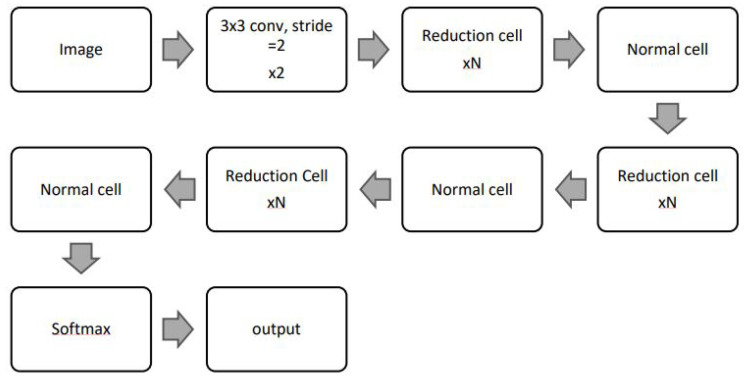
NASNetLarge architecture [31].

The NASNetLarge model, based on the Neural Architecture search network (NASNet), is designed using two cell types: normal cells and reduction cells. The default input size for NASNetLarge is 331 × 331. Details of the two cell types are shown in Figure 7.

#### 4.2.6. Xception

**Figure 8:**
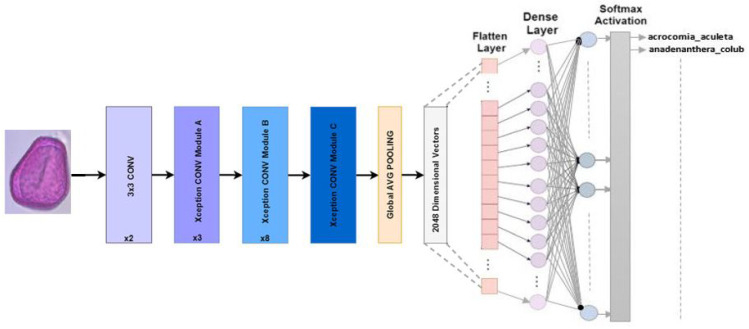
Multiclass classification is the goal of the Xception architecture.

The Keras library creator, Chollet, proposed adapting Inception architectures in 2016. Deep-separable convolutions are used to replace Inception modules in Xception. The ImageNet dataset resulted in Xception’s results exceeding those of InceptionV3 for accuracy in the Top-1 and Top-5. Xception has a parameter count that is approximately the same as that of InceptionV3, with around 23 million [30]. Our refined Xception model for Pollen73s classification is illustrated in Figure 8.

#### 4.2.7. DenseNet 201

**Figure 9:**
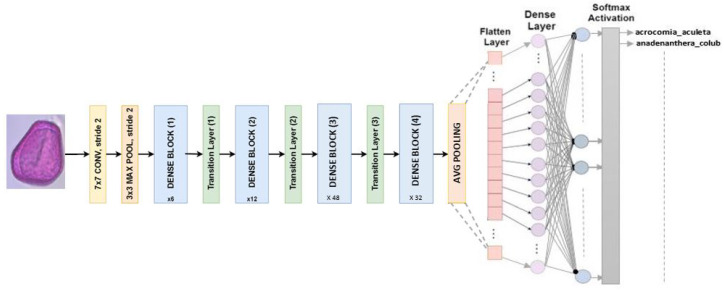
Multiclass classification is what DenseNet201 architecture is designed to do.

Has a fixed input size of 224 × 224 pixels for RGB images. DenseNet201 consists of 201 layers with over 19.9 million parameters. Dense blocks are divided into blocks where the dimensions of the feature maps stay the same, but the number of filters varies. Batch normalization is used for subsampling by transition layers, which are the input layers of the blocks [30]. In this study, our classifier is designed to replace the last fully connected layer with softmax activation, as shown in Figure 9.

#### 4.2.8. InceptionResNet V2

**Figure 10:**
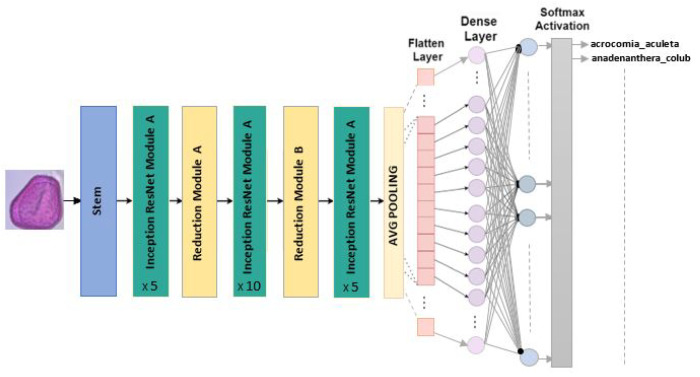
InceptionResNetV2 architecture for multiclass classification.

The fundamental module of this model is known as the Residual Origin Block. A 1 × 1 convolution layer is employed after each block to increase the size of the filter bank to match the input depth. Batch normalization is only applied to the traditional layers of this architecture. Featuring 164 layers deep and an image input dimension of 299×299, InceptionResNetV2 incorporates residual connections to blend convolutional filters of varying sizes. This use of residual connections avoids the degradation problems associated with deep networks and reduces training time [30]. Our refined model of InceptionResNetV2 for the classification of Pollen73s is illustrated in the Figure 10.

### 4.3. Holdout Cross-Validation Method

The holdout cross-validation method is a simple model validation method that consists of splitting the dataset into a training set and a test set. The principle of the method is as follows: the dataset is divided into two sets, the first of which is used to train the model and the second to test the model’s performance. The proportion for the training set is set at 80% and 20% for the test set [7,8].

### 4.4. Experimental Setup

- Data augmentation: In deep learning, this is a common way to increase the number of training data available to the model, which can enhance generalization and tackle underlearning. The various keras (https://keras.io/api/ accessed on 9 November 2022) parameters we have used are as follows:
  - rescale: 1./255, resize pixel values to between 0 and 1 by dividing each pixel by 255;
  - shear_range: 0.2, applies a random shear transformation to the image;
  - zoom_range: 0.2, randomly zooms the image in or out;
  - featurewise_center: True, subtracts the average of all training images from each image;
  - featurewise_std_normalization: True, divides each pixel by the standard deviation of the set of training images;
  - rotation_range: 20%, applies a 20% rotation to the image;
  - width_shift_range: 0.2, applies a random horizontal shift to the image;
  - height_shift_range: 0.2, applies a random vertical shift to the image;
  - horizontal_flip: True, applies random horizontal symmetry to the image;
  - vertical_flip: True, applies random vertical symmetry to the image;
  - validation_split: 0.2, separates 80% of training data and 20% for validation.
- Dropout layers: Typically, connecting all features directly to the Fully Connected (FC) layer can result in overfitting to the training dataset. Overfitting transpires when a model excels excessively on the training data, subsequently hindering its performance on new data. To mitigate this issue, a dropout layer is implemented, selectively removing a few neurons from the neural network during training to trim down the model’s complexity. Specifically with ResNet50, a dropout rate of 0.7 is utilized, leading to the random elimination of 70% of nodes from the neural network.
- Fine Tuning: This pertains to the notion of transfer learning, a machine learning methodology where knowledge gained from training for one specific problem is leveraged to train for another related task or domain. In transfer learning, the final layers of the pretrained network are removed, and new layers are trained for the intended task. This process was executed on the CNN InceptionResNetV2 architecture, with the batch size transitioning from 12 to 16 along with data augmentation. Truncation of the top layer was implemented for this model by introducing a new softmax layer fully connected to the top layer. Additionally, fine-tuning was conducted using the Adam (adaptive moment estimation) [32] optimizer and a learning rate of 0.001.
- Hyperparameters: These are parameters whose values are used to control the learning process. These parameters are obtained using grid search and random search. These methods optimize and sort the parameters of CNN models. These values can be adjusted to optimize model performance. Here are the hyperparameters used in the experimental setup (see Table 1).

### 4.5. Performance Evaluation Measures

Confusion matrix, precision, recall, $F1_{score}$ and accuracy are the standard classification model evaluation methods.

**Figure 11:**
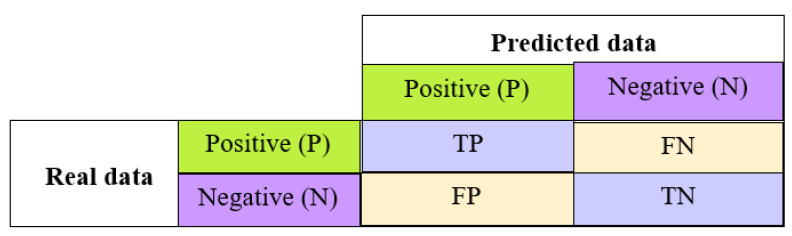
Confusion matrix.

- Confusion matrix: This entails a comprehensive evaluation of the model’s performance, involving four key metrics: true positive (TP), false positive (FP), false negative (FN) and true negative (TN). These metrics are organized in a tabular format with rows and columns, where the rows portray the actual classes and the columns depict the predicted classes (see Figure 11).
- Precision: This indicates the percentage of cases that were correctly identified out of all identified cases.
  (1) Precision,P=TP/(TP+FP),0%≤Precision≤100%.
- Recall: This is the number of cases correctly identified among all positive representations. It measures a model’s ability to identify all positive results.
  (2) Recall,R=TP/(TP+FN),0%≤Recall≤100%.
- $F1_{score}$: This is the harmonic mean of precision and recall. It provides a compromise between precision and recall.
  (3) $$F1_{score},F1=2\times \left(P\times R\right)/\left(P+R\right),0\%\leq F1_{score}\leq 100\%.$$
- Accuracy: This is the ratio of correct predictions to the total number of observations (total input samples). However, this measure is not very reliable if the classes are unbalanced.
  (4) Accuracy,Acc=TP+TN/(FN+TP+TN+FP),0%≤Accuracy≤100%.

## 5. Results and Discussion

### 5.1. Architectural Performance Summary Table

Table 2 shows the different results obtained by the eight models.

We managed to achieve the highest precision and accuracy, 97.5545% and 97.202%, using DenseNet201 architecture and a batch size of 16. Convolutional neural networks like DenseNet201, InceptionResNetV2 and ResNet50 demonstrated superior performance with a batch size of 16. VGG19, VGG16 and Xception attained optimal results with a batch size of 8, while InceptionV3 and NASNet accomplished this with batch sizes of 4 and 12, respectively. Notably, DenseNet201 exhibited a remarkable $F1_{score}$ of 97.26%, indicating well-balanced true positives, true negatives, false positives and false negatives, leading to a notable accuracy rate of 97.217%. Significantly, ResNet50 yielded outcomes that diverged from those of DenseNet201, exhibiting a precision 2.50% lower, aligning more closely with InceptionV3 with a difference of 0.818% in precision. Conversely, VGG19 displayed the least favorable results in comparison to other CNNs, whereas InceptionResNetV2, VGG16, NASNet and Xception produced comparable results. The best performances were characterized by the values of the metrics highlighted in bold. The significance of the accuracy rates reached by DenseNet201 and ResNet50, standing at 97.217% and 94.257%, respectively, lies in the diverse nature of pollen types within POLLEN73S. Notably, Khanzhina et al. [33] accomplished a 99% accuracy level in a dataset comprising 5 pollen types, which diminished to 95% as the dataset expanded to include 11 pollen types, underscoring the challenge of maintaining high accuracy rates when dealing with a larger array of pollen types. Drawing a comparison, Sevillano and Aznarte [16] employed three CNNs to categorize 23 pollen types within the POLEN23E [20] dataset and achieved a commendable 97% accuracy rate. The POLLEN23E dataset has a total of 23 pollen grain types and POLLEN73S has 22 of them. The performance of DenseNet201 and ResNet50 on POLLEN73S was satisfactory, as shown by this.

### 5.2. CNN Architectures’ Performances

**Figure 12:**
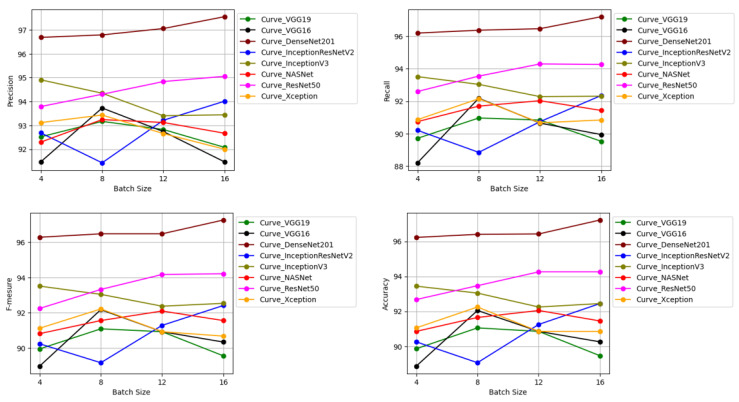
Performances of eight architectures in terms of (**top left**) precision, (**top right**) recall, (**bottom left**) $F1_{score}$, (**bottom right**) accuracy.

Figure 12 depicts CNN architectures’ performances.

We found that for all evaluation measures, DenseNet201 architecture performs better on all batch sizes.

### 5.3. Performance Comparison of CNN Architectures for Pollen Grain Classification: Current Results Compared to [4]

In Table 3, we summarize the results obtained for POLLEN73S classification by the following two methods: the holdout cross-validation method (current works) in the first column of each metric and the five-block cross-validation method of [4] in the second column of each metric.

Performance graphs for the eight architectures are displayed in Appendix A while graphs of pollen73S class probabilities when predicting a pollen grain using the best-performing architecture (DenseNet201) are displayed in Appendix B.

## 6. Conclusions

The aim of this work was to classify pollen grains from an annotated image dataset called POLLEN73S, and improve the performance of eight state-of-the-art convolutional neural networks (CNNs) already used in existing work, for the classification of pollen grains from the Brazilian savannah (Cerrado).

We used the holdout cross-validation method with hyperparameters and techniques such as dropout, data augmentation and fine-tuning, in contrast to the five-block cross-validation method used in previous works. The DenseNet201 and ResNet50 architectures outperformed the other CNNs tested, achieving performances of 97.217% and 94.257%, respectively. Compared to the best results obtained before, our results are therefore relatively appreciable.

In future work, we plan to further explore CNN hyperparameters. We also intend to better explore the VGG19 architecture to find a convolutional architecture specific to the POLLEN73S dataset, because even using the architecture defined for the ImageNet dataset, this architecture worked well in the case of the present work. Additionally, we plan to combine different CNNs in order to continue to improve performance. 

## Figures and Tables

**Table 1:** Hyperparameters for different models.

Parameters	Parameter Values
batch size	4, 8, 12 and 16
optimizer	Adam and RMSprop
lr(learning rate)	0.001, 0.00001 (min_lr = 0.0001)
	and 0.00003 (min_lr = 0.0003)
betas($\beta_{1}$ and $\beta_{2}$)	[0.9,0.999]
eps(ϵ)	$1\times 10^{-7}$
weight decay	0
momentum	0.0
ema_momentum	0.99

**Table 2:** Performance summary of the different architectures used.

CNN Models	Batch Size	Precision	Recall	$F1_{\mathrm{score}}$	Accuracy (Loss)
VGG19	4	92.519	89.713	89.941	89.861 (0.3608)
	8	**93.164**	**90.966**	**91.083**	**91.054 (0.3512)**
	12	92.824	90.839	90.920	90.855 (0.3453)
	16	92.075	89.528	89.550	89.463 (0.3989)
VGG16	4	91.471	88.205	88.963	88.867 (0.3635)
	8	**93.726**	**92.180**	**92.172**	**92.048 (0.3154)**
	12	92.737	90.648	90.920	90.855 (0.3183)
	16	91.466	89.945	90.333	90.258 (0.3755)
DenseNet201	4	96.686	96.193	96.282	96.223 (0.2232)
	8	96.790	96.368	96.477	96.400 (0.17)
	12	97.057	96.462	96.477	96.421 (0.1918)
	16	**97.554**	**97.202**	**97.260**	**97.217 (0.1352)**
InceptionResNetV2	4	92.681	90.207	90.235	90.258 (0.8402)
	8	91.428	88.849	89.159	89.066 (0.5553)
	12	93.212	90.739	91.279	91.252 (0.4930)
	16	**94.012**	**92.352**	**92.407**	**92.445 (0.5578)**
InceptionV3	4	**94.906**	**93.511**	**93.509**	**93.439 (0.5030)**
	8	94.346	93.035	93.040	93.042 (0.4374)
	12	93.400	92.281	92.368	92.247 (0.4073)
	16	93.441	92.309	92.531	92.445 (0.3779)
NASNet	4	92.297	90.742	90.809	90.855 (0.5790)
	8	93.240	91.696	91.553	91.650 (0.5016)
	12	**93.120**	**92.030**	**92.094**	**92.048 (0.4452)**
	16	92.667	91.429	91.553	91.451 (0.4603)
ResNet50	4	93.788	92.596	92.239	92.673 (0.2995)
	8	94.301	93.542	93.319	93.465 (0.3405)
	12	94.834	94.292	94.168	94.257 (0.2869)
	16	**95.054**	**94.260**	**94.210**	**94.257 (0.2119)**
Xception	4	93.115	90.862	91.115	91.054 (0.5123)
	8	**93.431**	**92.149**	**92.211**	**92.247 (0.3052)**
	12	92.655	90.670	90.920	90.855 (0.4729)
	16	91.998	90.841	90.670	90.855 (0.3102)

**Table 3:** Synthesis between current results and [4] results for the eight architectures. [P = Precision, R = Recall, F = $F1_{score}$, A = Accuracy, Aut = Authors [4].

CNN Models	Batch Size	P	P-Aut	R	R-Aut	F	F-Aut	A	A-Aut
VGG19	4	92.519	**89.9**	89.713	**89.2**	89.941	**91.5**	89.861	**89.7**
	8	**93.164**	89.0	**90.966**	88.5	**91.083**	90.7	**91.054**	88.9
	12	92.824	89.3	90.839	88.8	90.920	91.0	90.855	89.1
	16	92.075	87.7	89.528	87.3	89.550	90.1	89.463	87.7
VGG16	4	91.471	87.6	88.205	87.0	88.963	89.5	88.867	87.4
	8	**93.726**	87.4	**92.180**	86.8	**92.172**	89.7	**92.048**	87.1
	12	92.737	**88.6**	90.648	**88.1**	90.920	**90.1**	90.855	**88.4**
	16	91.466	88.0	89.945	87.5	90.333	90.0	90.258	87.9
DenseNet-201	4	96.686	87.9	96.193	87.0	96.282	89.3	96.223	87.8
	8	96.790	95.6	96.368	95.6	96.477	96.3	96.4	95.7
	12	97.057	**95.7**	96.462	**95.7**	96.477	**96.4**	96.421	**95.8**
	16	**97.554**	95.3	**97.202**	95.1	**97.260**	95.9	**97.217**	95.2
InceptionResNet-V2	4	92.681	74.4	90.207	71.8	90.235	78.8	90.258	74.0
	8	91.428	88.6	88.849	88.1	89.159	90.9	89.066	88.6
	12	93.212	**89.7**	90.739	**89.4**	91.279	**91.3**	91.252	**89.7**
	16	**94.012**	89.6	**92.352**	89.3	**92.407**	90.9	**92.445**	89.6
Inception-V3	4	**94.906**	72.7	**93.511**	70.7	**93.509**	76.2	**93.439**	72.5
	8	94.346	87.8	93.035	86.9	93.040	89.0	93.042	87.6
	12	93.400	**90.3**	92.281	**90.0**	92.368	**91.6**	92.247	**90.4**
	16	93.441	89.9	92.309	89.5	92.531	91.2	92.445	89.9
NASNet	4	92.297	76.8	90.742	77.0	90.809	81.7	90.855	77.0
	8	93.240	86.2	91.696	85.9	91.553	87.9	91.650	85.9
	12	**93.120**	**87.4**	**92.030**	**87.4**	**92.094**	**89.8**	**92.048**	**87.4**
	16	92.667	86.6	91.429	86.5	91.553	89.1	91.451	86.5
ResNet-50	4	93.788	85.0	92.596	85.0	92.239	88.0	92.673	85.0
	8	94.301	93.0	93.542	93.0	93.319	94.0	93.465	93.0
	12	94.834	**94.0**	94.292	**95.0**	94.168	**95.0**	94.257	**95.0**
	16	**95.054**	93.9	**94.260**	94.0	**94.210**	94.9	**94.257**	94.0
Xception	4	93.115	85.0	90.862	85.0	91.115	86.0	91.054	85.0
	8	**93.431**	90.0	**92.149**	90.0	**92.211**	91.0	**92.247**	90.0
	12	92.655	**90.1**	90.670	**90.1**	90.920	**92.0**	90.855	**90.1**
	16	91.998	90.0	90.841	90.0	90.670	91.0	90.855	90.0

**Figure A1:**
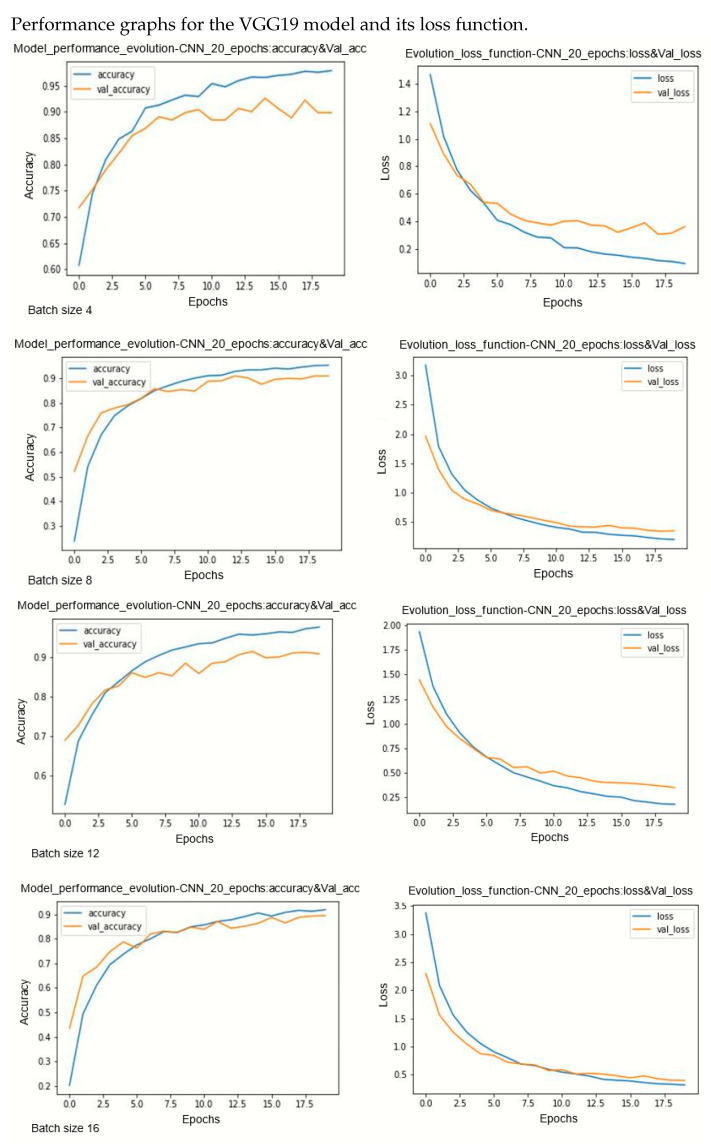
Performance evolution and losses of Vgg19.

**Figure A2:**
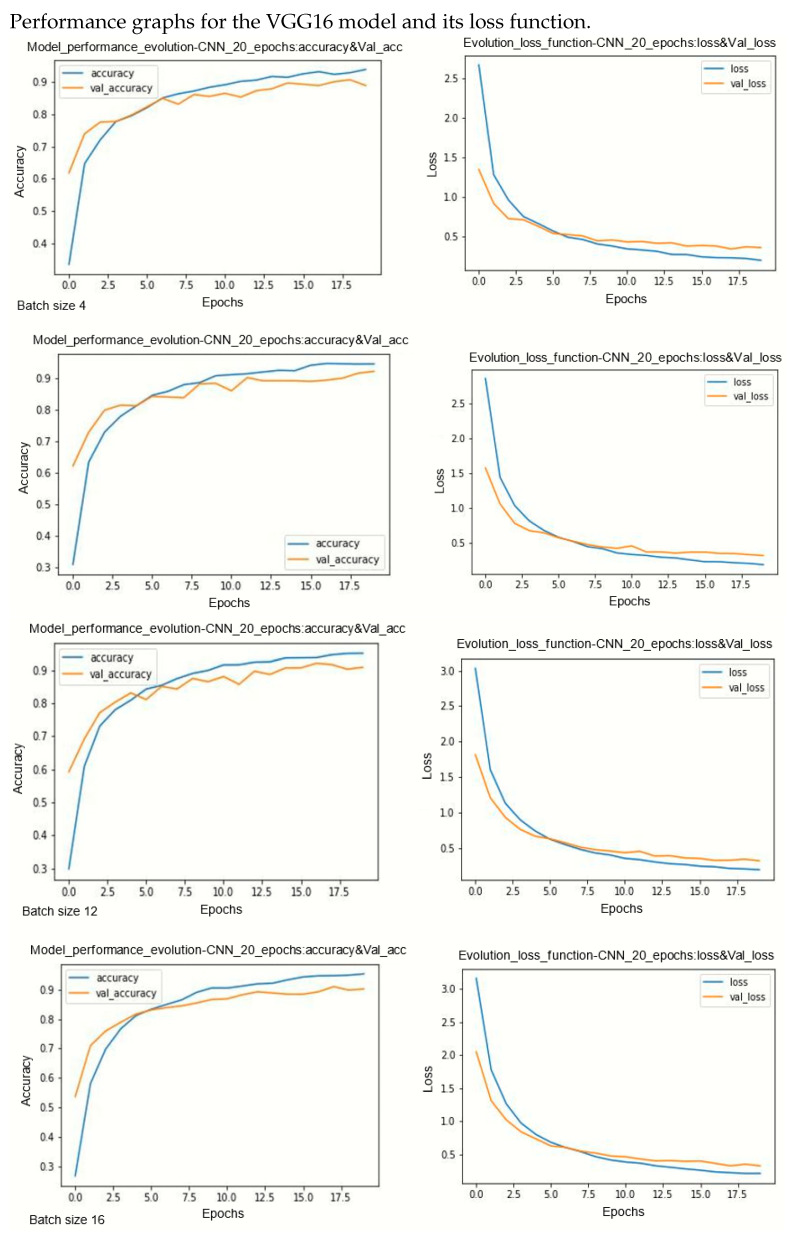
Performance evolution and losses of Vgg16.

**Figure A3:**
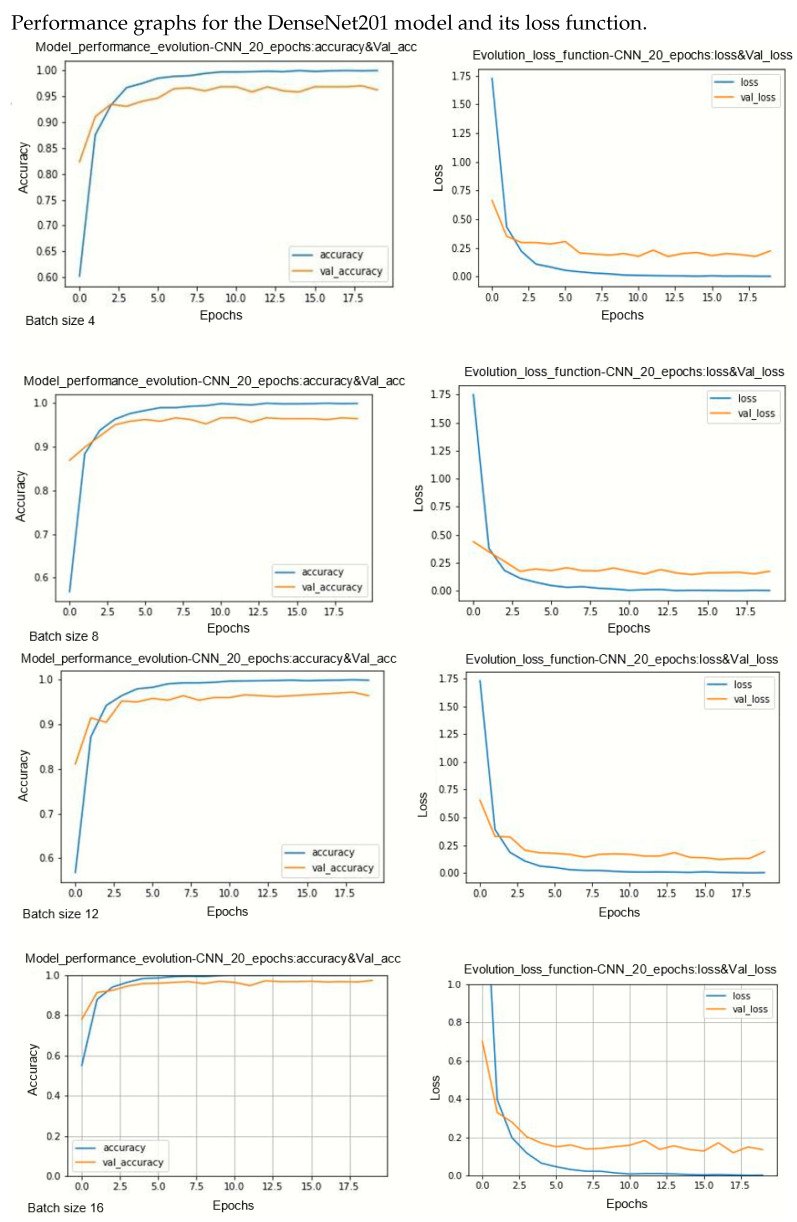
Performance evolution and losses of DenseNet201.

**Figure A4:**
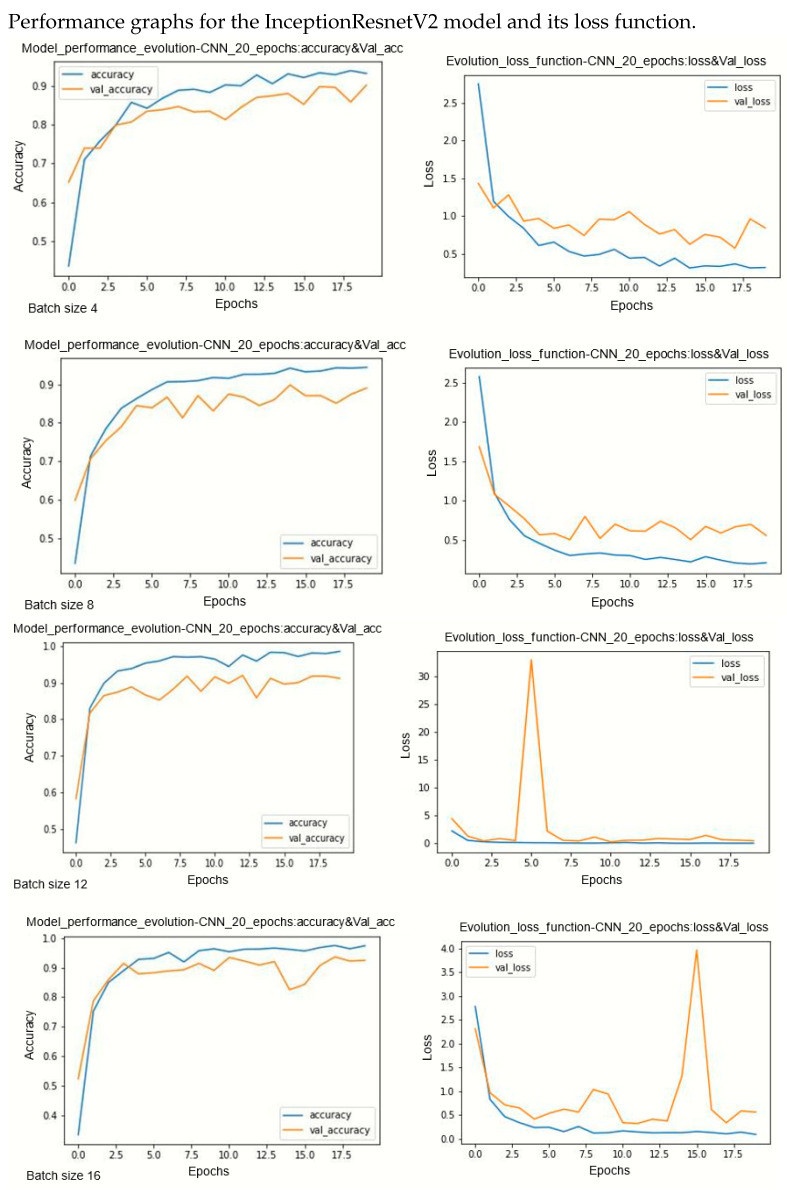
Performance evolution and losses of InceptionResnetV2.

**Figure A5:**
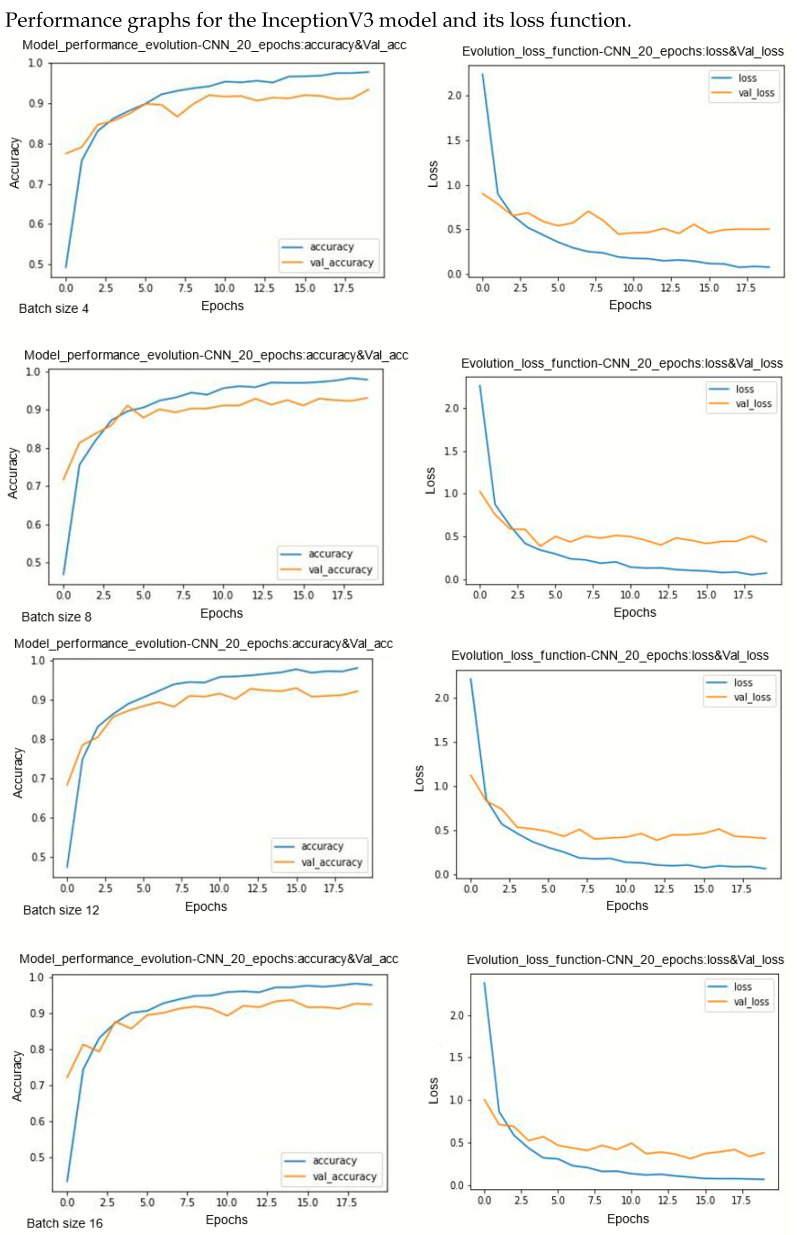
Performance evolution and losses of InceptionV3.

**Figure A6:**
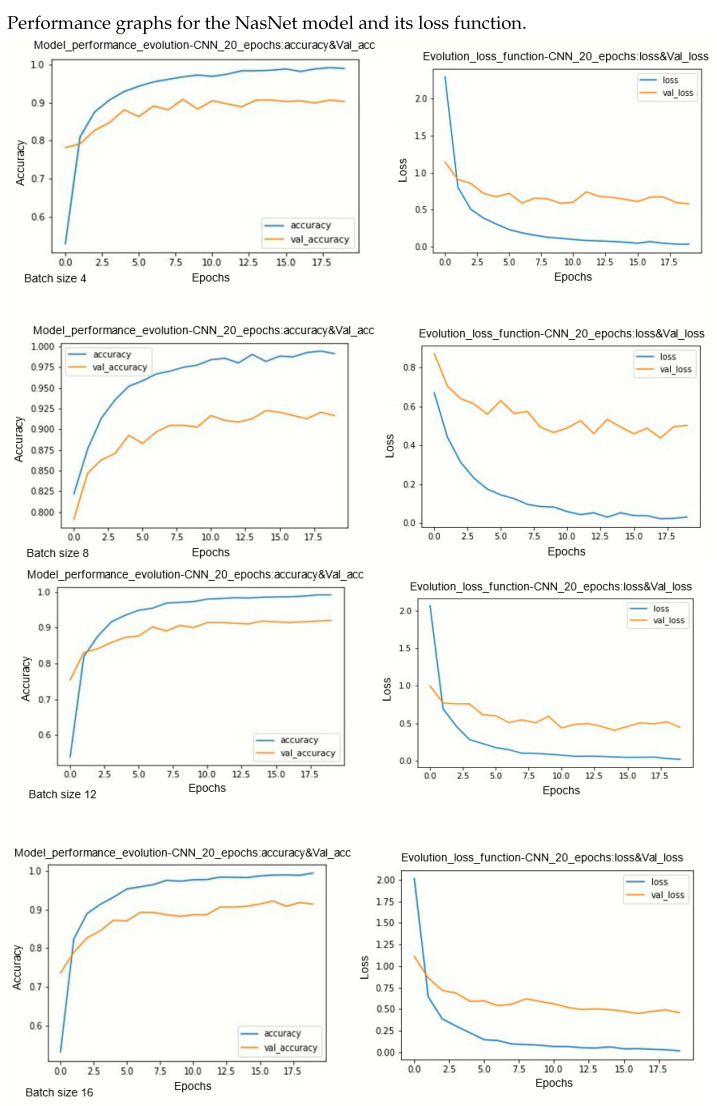
Performance evolution and losses of NasNet.

**Figure A7:**
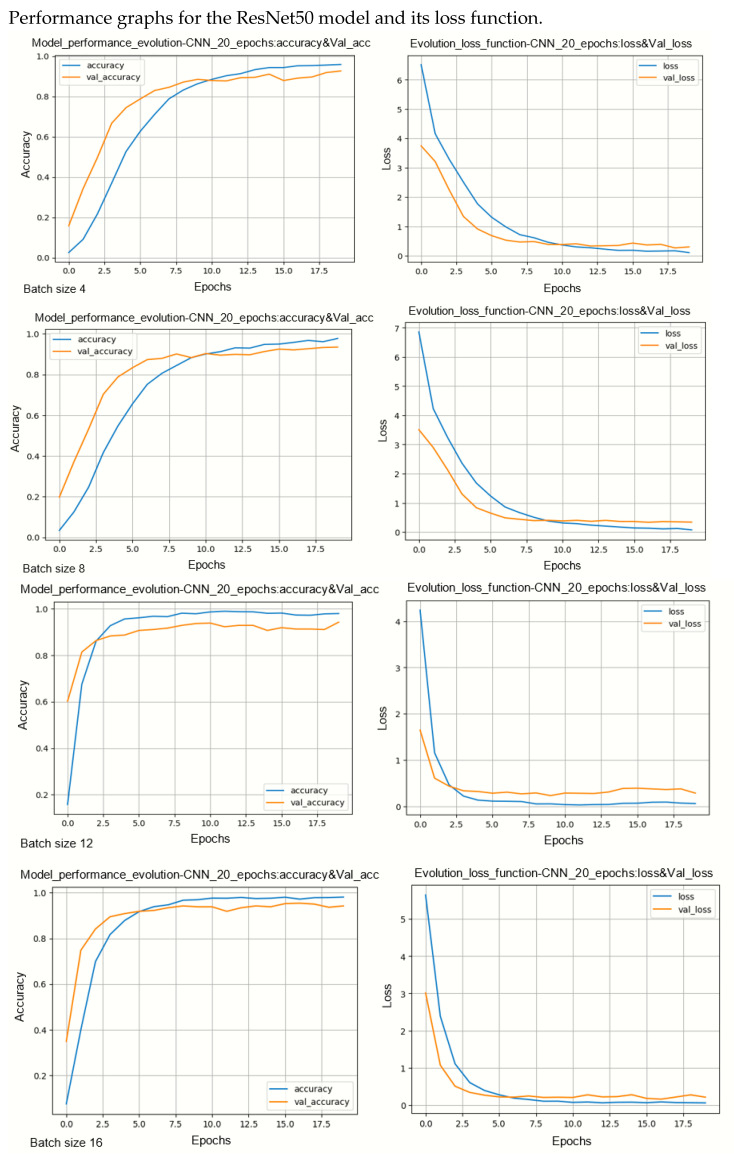
Performance evolution and losses of ResNet50.

**Figure A8:**
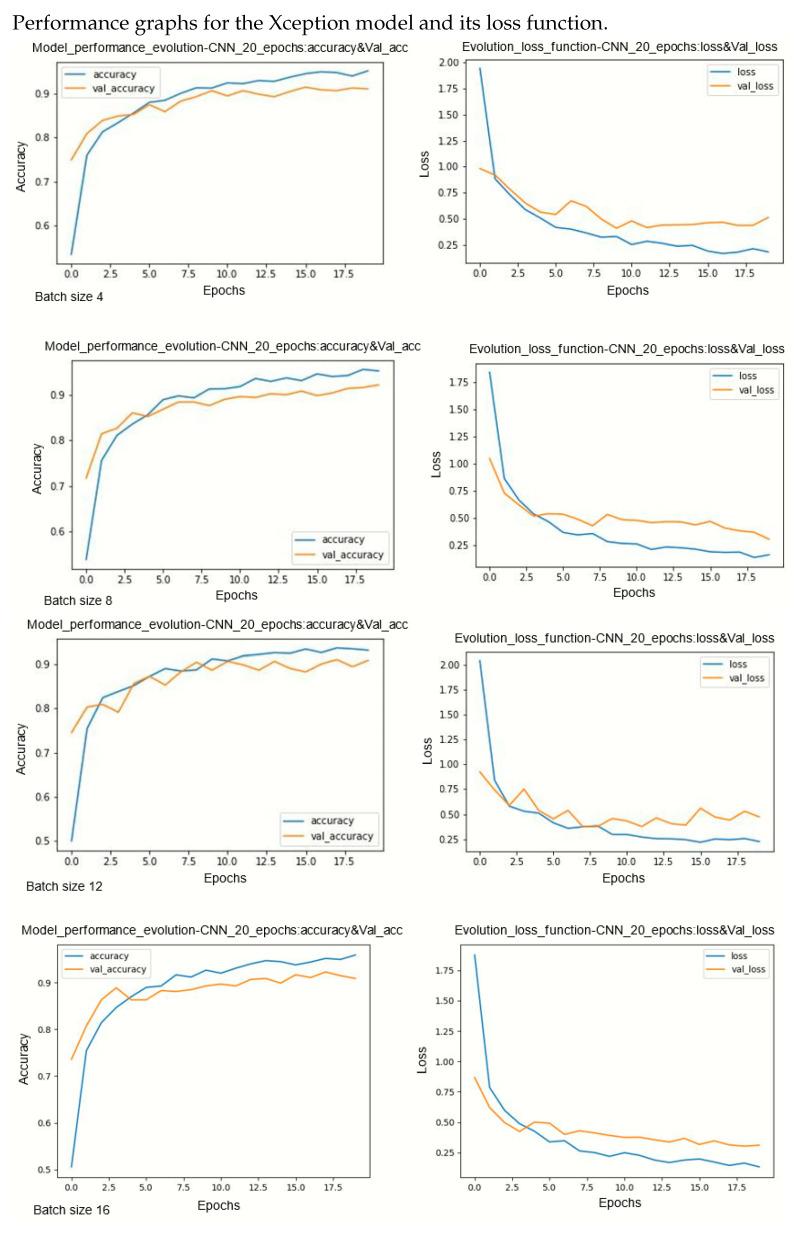
Performance evolution and losses of Xception.

**Figure A9:**
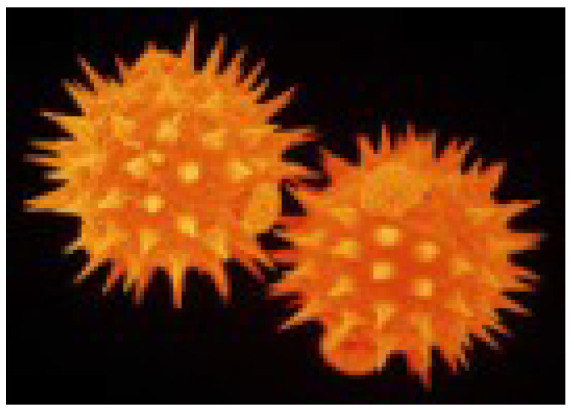
Image to predict (https://gapp.org/your-pollinator-garden-online/ accessed on 10 November 2023).

**Figure A10:**
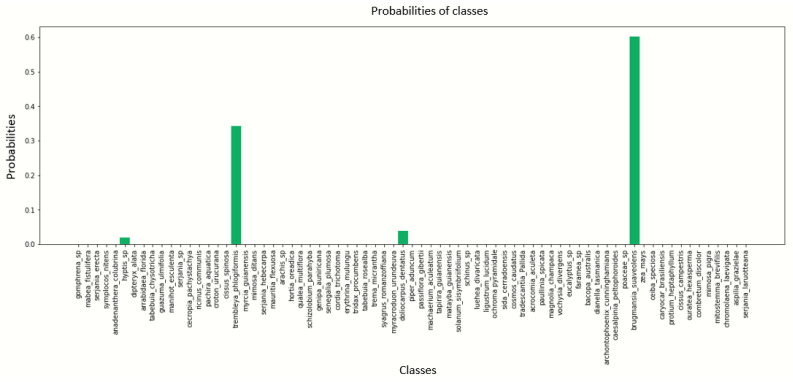
Pollen image prediction using the DenseNet201 architecture with a batch size of 4.

**Figure A11:**
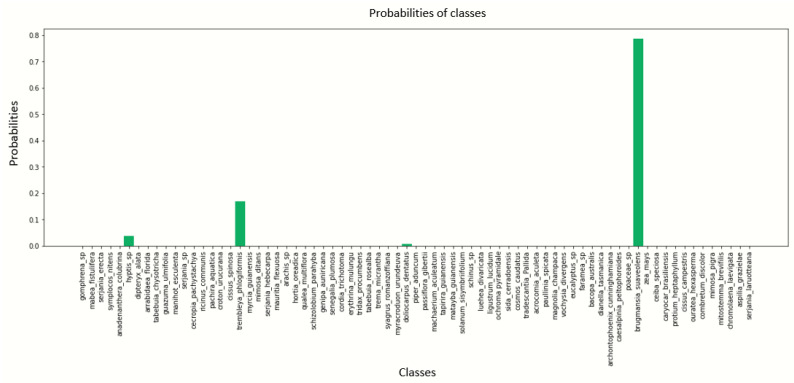
Pollen image prediction using the DenseNet201 architecture with a batch size of 8.

**Figure A12:**
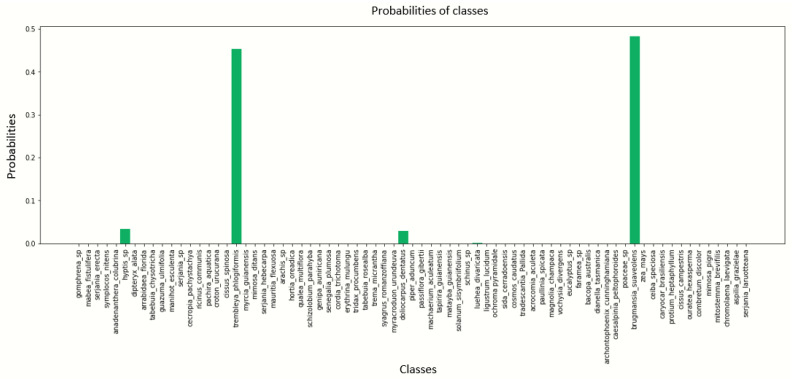
Pollen image prediction using the DenseNet201 architecture with a batch size of 12.

**Figure A13:**
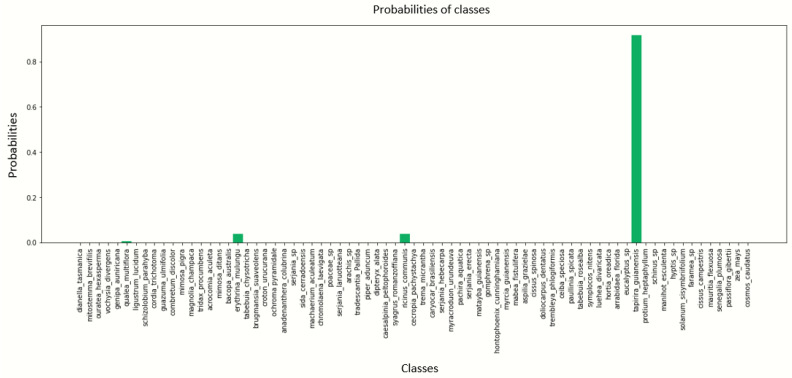
Pollen image prediction using the DenseNet201 architecture with a batch size of 16.

## Data Availability

Data used in this work can be found in Astolfi et al. in [4].

## Appendix A. Performance Graphs for the Eight Architectures

This annex illustrates the training and validation performance as well as loss trends of the models detailed in Section 4.2 (VGG19, VGG16, DenseNet201, InceptionResNetV2, InceptionV3, NASNet, ResNet50, Xception) with varying batch sizes of 4, 8, 12, and 16. These results were gathered during the training and testing phases of the models over 20 epochs (iterations). All architectures were trained with accuracy as the overall evaluation metric.

During model training, two primary challenges are often encountered: underfitting and overfitting. Underfitting occurs when there is a significant disparity between the training loss curve and the validation loss curve. This discrepancy indicates that the model is not effectively learning from the training data. On the other hand, overfitting occurs when the validation error surpasses the training error as the number of epochs increases. The loss curves provide insights into the model’s progression towards its target state across epochs, with lower loss values indicating a more efficient model. By observing several curves, two observations can be made. Models are less sensitive on underlearning and overlearning.

## Appendix B. Graphs of pollen73S Class Probabilities When Predicting a Pollen Grain Using the Best-Performing Architecture (DenseNet201)

Appendix B illustrates the prediction probabilities of each class derived from the performance of a CNN architecture used to classify POLLEN73S from 20 epochs and batch sizes of 4, 8, 12 and 16, respectively (see Figure A9, Figure A10, Figure A11, Figure A12 and Figure A13).
